# Gut microbiota changes in patients with Alzheimer’s disease spectrum based on *16S rRNA* sequencing: a systematic review and meta-analysis

**DOI:** 10.3389/fnagi.2024.1422350

**Published:** 2024-08-08

**Authors:** Hui Li, Xiaopan Cui, Yuxiu Lin, Fengqiong Huang, Ayong Tian, Rongwei Zhang

**Affiliations:** ^1^Department of Gerontology and Geriatrics, The First Affiliated Hospital of China Medical University, Shenyang, Liaoning, China; ^2^Department of Anesthesiology, The Fourth Affiliated Hospital of China Medical University, Shenyang, Liaoning, China

**Keywords:** Alzheimer’s disease, gut microbiota, *16S rRNA* sequencing, biomarkers, meta-analysis

## Abstract

**Background:**

The gut microbiota (GM) is hypothesized to play roles in Alzheimer’s disease (AD) pathogenesis. In recent years, many GM composition and abundance investigations in AD patients have been conducted; however, despite this work, some results remain controversial. Therefore, we conducted a systematic review and meta-analysis using 16S ribosomal RNA (*16S rRNA*) sequencing to explore GM alterations between patients with AD spectrum and healthy controls (HCs).

**Methods:**

A systematic and comprehensive literature search of PubMed, Web of Science, Embase, the Cochrane Library, China National Knowledge Infrastructure, China Biology Medicine disc database, WanFang database and Social Sciences Citation Index databases was conducted from inception to January 2023. Inclusion and exclusion criteria were strictly defined, and two researchers independently screened and extracted information from selected studies. Data quality were evaluated according to the “Cochrane system evaluator manual” and pooled data were comprehensively analyzed using Stata 14 software with standardized mean differences (SMDs) and 95% confidence intervals (95% CIs) used to measure effect sizes. Also, geographical heterogeneity effects (related to cohorts) on GM abundance were examined based on subgroup meta-analyses if sufficient studies reported outcomes. Finally, publication bias was assessed using funnel plots.

**Results:**

Out of 1566 articles, 13 studies involving 581 patients with AD spectrum and 445 HCs were deemed eligible and included in our analysis. In summary, a decreased microbiota alpha diversity and a significantly distinct pattern of clustering with regard to beta diversity were observed in AD spectrum patients when compared with HCs. Comparative analyses revealed a decreased *Ruminococcus*, *Faecalibacterium*, *Lachnospira*, *Dialister*, *Lachnoclostridium*, and *Roseburia* abundance in AD spectrum patients while *Phascolarctobacterium*, *Lactobacillus*, and *Akkermansia muciniphila* were more enriched in patients when compared to HCs. Furthermore, regional variations may have been in play for intestinal microbes such as *Bacteroides*, *Bifidobacterium*, and *Alistipes*.

**Conclusion:**

Our meta-analysis identified alterations in GM abundance in patients with AD spectrum, with 12 genera from four major phyla significantly associated with AD. Moreover, we provided evidence for region-specific alterations in *Bacteroides*, *Bifidobacterium*, and *Alistipes* abundance. These findings may have profound implications for the development of innovative GM-based strategies to prevent and treat AD.

**Systematic review registration:**

https://doi.org/10.37766/inplasy2024.6.0067, identifier INPLASY202460067.

## Introduction

Alzheimer’s disease (AD) is a neurodegenerative disorder characterized by progressive cognitive and memory decline, making it the predominant dementia type. With global aging trends, the number of individuals affected by dementia is projected to reach 78 million by 2030 (Gauthier et al., 2021). In recent years, AD has emerged as a significant public health concern impacting overall wellbeing and quality of life metrics across global populations (Alzheimer’s, 2023). Therefore, it is crucial to diagnose, prevent, and treat AD during its early or even preclinical stages.

AD pathophysiological mechanisms and clinical manifestations suggest that the disease should be conceptualized as a continuum or spectrum, encompassing three distinct phases: preclinical AD (including subjective cognitive decline, SCD), mild cognitive impairment (MCI), and Dementia of Alzheimer’s type, also known as AD (Fernandes et al., 2020; Alzheimer’s, 2023). The characteristic histopathological hallmark of AD is the extraneuronal aggregation of amyloid-β (Aβ) peptide fragments into plaques, along with intraneuronal abnormal tau protein accumulation, which both serve as AD biomarkers (Long and Holtzman, 2019). Although no cure exists for AD, recent evidence has highlighted the potential role of the gut microbiota (GM) in disease development or exacerbation (Bonfili et al., 2018; Kim et al., 2020). Furthermore, several studies have reported promising results in terms of enhancing cognition via non-pharmacological interventions such as fecal microbial transplantation (Sun et al., 2019) and probiotics at early disease stages (Hwang et al., 2019).

The GM is a complex and abundant microbial community in the human gastrointestinal tract, and has important physiological roles regulating immune function, promoting food catabolism and metabolism, secreting metabolites, and limiting pathogen abundance (Rolhion and Chassaing, 2016). Numerous studies have confirmed the significant involvement of the GM in the occurrence and development of central nervous system diseases, such as anxiety disorders (Cox and Weiner, 2018), autism (Finegold et al., 2010), depression (Valles-Colomer et al., 2019), and Parkinson’s disease (PD) (Scheperjans et al., 2015). Recently, several investigations reported a correlation between intestinal flora and AD, suggesting altered GM composition and abundance among AD patients. Evidence has also indicated that neuroinflammation plays fundamental roles in AD onset and progression, while activation of the human innate immune system by gut microorganisms may drive brain inflammation (Cheng et al., 2019; Kowalski and Mulak, 2019; Sochocka et al., 2019). Gut microorganisms secrete increased amyloid protein and lipopolysaccharide (LPS) that modulate signaling pathways to potentially increase amyloid aggregation, and lead to inflammation via inflammatory cytokine production and immunogenic changes within the brain (Hill and Lukiw, 2015; Taylor and Matthews, 2015; Sochocka et al., 2019). Emerging studies indicate that metabolites generated by GM play a role in regulating the differentiation, maturation, and activation of reactive astrogliosis and microgliosis, which provide support and protection to neurons, clearing dead cells and foreign particles to maintain homeostasis in the brain. However, intestinal dysbiosis abnormally activates glial cells, potentially propagating Aβ toxicity, increasing Aβ accumulation, or releasing proinflammatory cytokines and reactive oxygen species, all of which are harmful to neurons, facilitate tau pathology, and further exacerbate inflammation, leading to neuronal damage and the progression of AD pathology (Yang and David, 2018; Ashley et al., 2022).

Recently, GM exploration as a biomarker and potential target to prevent and treat AD has gained popularity due to simple specimen acquisition, easy detection, and targeted regulation. In bacteria, 16S ribosomal RNA (*16S rRNA*) corresponds to the DNA sequence encoding rRNA, which is universally present in bacterial genomes (Gao et al., 2021). *16S rRNA*-based sequencing is a rapid, cost-effective, and minimal labor-intensive approach for microbial detection; it allows for the comprehensive analysis of whole microbial composition and significantly enhances bacterial identification and resolution (Forde and O’Toole, 2013). Thus, in recent years, *16S rRNA* sequencing techniques have emerged as predominant methods for investigating microbial ecosystem composition. To date, several clinical studies (Ling et al., 2020; Guo et al., 2021; Liu et al., 2021; Kaiyrlykyzy et al., 2022; Ferreiro et al., 2023) have used gut flora *16S rRNA* high-throughput sequencing to analyze GM relative abundance in patients with AD. However, due to variations in bacterial detection techniques and inconsistent results from *16S rRNA* sequencing, accurate and specific comparisons across studies are often limited. Several observational studies have found inconsistencies in the composition and diversity of GM associated with AD. For instance, Guo et al. (2021) identified genera such as *Prevotella*, *Bacteroides*, and *Lachnospira* as differing between AD patients and HCs, while Liu et al. (2021) found differences in genera including *Blautia*, *Bacteroides*, and *Ruminococcus*. Meantime, Guo et al. (2021) reported a higher relative abundance of *Bacteroides* in AD patients, whereas (Liu et al., 2019) arrived at the opposite conclusion. Additionally, Sheng et al. (2021) observed a trend toward a gradual decrease in GM abundance and diversity from individuals with SCD to MCI. However, a meta-analysis including AD and MCI patients indicated a slight increase in GM diversity and abundance among MCI patients. In this study, we conducted a systematic review and meta-analysis to investigate altered GM abundance in AD spectrum patients (i.e., SCD, MCI, and AD) based on *16S rRNA* sequencing.

## Materials and methods

The systematic review was performed according to the PRISMA guidelines (Preferred Reporting Items for Systematic Reviews and Meta-Analyses), and the protocol was registered at INPLASY (International Platform for Registered Protocols for Systematic Reviews and Meta-Analyses) under registration number INPLASY202460067.^1^

### Search strategy

To identify information/data related to intestinal microbial profiles associated with AD, an extensive search was conducted across different medical databases, including PubMed, Web of Science, Embase, the Cochrane Library, China National Knowledge Infrastructure, China Biology Medicine disc database, WanFang database and Social Sciences Citation Index databases from inception to January 2023. The completed search strategy for Embase was: (“rna, ribosomal, 16s”/exp OR “16s rrna”:ab,ti OR “rrna 16s”:ab,ti OR “16s ribosomal rna”:ab,ti OR “rna, 16s ribosomal”:ab,ti OR “ribosomal rna, 16s”:ab,ti OR “16s ribosome rna”:ab,ti OR “16s rdna”:ab,ti OR “rdna 16s”:ab,ti OR “16s ribosomal dna”:ab,ti OR “dna, 16s ribosomal”:ab,ti OR “ribosomal dna, 16s”:ab,ti OR “16s ribosome dna”:ab,ti OR “16s rrna gene”:ab,ti) AND (“alzheimer disease”/exp OR “dementia”/exp OR alzheimer:ab,ti OR “alzheimers disease”:ab,ti OR dementia:ab,ti OR cognitive:ab,ti OR cogntion:ab,ti OR amentia: ab,ti OR mci:ab,ti OR “mild cognitive impairment”:ab,ti OR “mild cognitive defect”:ab,ti OR scd:ab,ti OR “subjective cognitive decline”:ab,ti OR “subjective cognitive impairment”:ab,ti). Furthermore, we also reviewed cited references of the retrieved articles to identify additional published and unpublished studies.

### Inclusion and exclusion criteria

Inclusion criteria were as follows: (1) AD spectrum patients diagnosed using validated criteria (Diagnostic and Statistical Manual of Mental Disorders or National Institute on Aging and Alzheimer’s Association guidelines) (First et al., 2002; Hyman et al., 2012); (2) GM comparisons conducted between AD spectrum and HCs using *16S rRNA* sequencing; (3) GM samples came from stool samples; (4) Accessible raw data were, such as relative GM abundance at distinct levels, microbial composition, community structures, and diversity indices; (5) Studies including randomized controlled trials (RCTs), cohort studies, and case-control studies; and (6) Studies not limited by language type (i.e., English, Chinese).

Exclusion criteria were as follows: (1) Studies without HCs; (2) Microbial analyses using other microbial detection methods; (3) Ambiguous literature and corresponding data and results that could not be extracted; (4) Studies with insufficient or overlapping data (the most recent and complete data were chosen); and (5) Systematic reviews, animal studies, conference abstracts, case reports, and commentaries.

### Study selection and data extraction

Using our retrieval strategy, two independent investigators extracted data and discussed any post-extraction discrepancies. Disagreements were resolved by discussion with a third reviewer. If necessary, the corresponding authors of selected studies were contacted (e-mail) to request any missing data. Data collected from selected studies included: general information (first author and publication year, study design, and region), participant characteristics (sample size, age, and sex ratio), adjusted confounding factors, and information pertaining to GM alterations (relative GM abundance by taxonomic, microbial composition, community structure levels, and diversity index).

### Risk of bias and quality assessment

Based on extracted data, the Cochrane Risk of Bias Assessment Tool (Higgins et al., 2011) was used for RCTs which had five domains: randomization, deviation from intervention, missing data, outcome measurements, and selective reporting. The Newcastle–Ottawa scale (Wells et al., 2000) was used for observational/non-randomized studies with domain selection, comparability, and exposure/outcomes.

### Statistical analysis

This meta-analysis was conducted using Stata 14.0 software. Standardized mean differences (SMD) and 95% confidence intervals (CI) came from fixed effects or random effects models for quantitative synthesis. Heterogeneity was evaluated using the Cochran Q statistic and quantified by *I*^2^ tests, with an *I*^2^ > 50% indicating moderate-to-high heterogeneity (Higgins et al., 2003). Subgroup analysis was performed to investigate sources of heterogeneity, while sensitivity analyses conducted if necessary. Publication bias was visually assessed using funnel plots (Sterne et al., 2011).

## Results

### Literature searches and study characteristics

**FIGURE 1:**
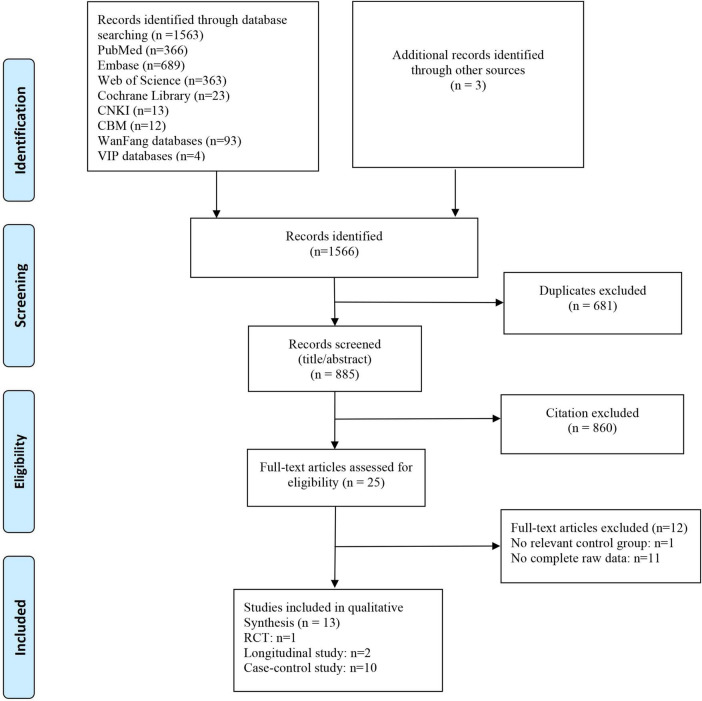
Flow chart of the evidence search and selection process.

The initial search yielded 1,566 articles. After excluding 681 duplicates, two independent reviewers assessed titles and abstracts to exclude 860 irrelevant articles. Subsequently, full-text reviews were conducted on 25 articles, and finally 13 studies were included in the meta-analysis (Figure 1). Studies comprised 10 case-control (Vogt et al., 2017; Zhuang et al., 2018; Li et al., 2019; Liu et al., 2019, 2021; Ling et al., 2020; Guo et al., 2021; Sheng et al., 2021; Zhou et al., 2021; Khedr et al., 2022) and two longitudinal studies (Haran et al., 2019; Zhang et al., 2021), and also one RCT study (Nagpal et al., 2019). The geographical location of studies included China (Zhuang et al., 2018; Li et al., 2019; Liu et al., 2019, 2021; Ling et al., 2020; Guo et al., 2021; Sheng et al., 2021; Zhang et al., 2021; Zhou et al., 2021), USA (Vogt et al., 2017; Haran et al., 2019; Nagpal et al., 2019), and Egypt (Khedr et al., 2022). All studies used *16SrRNA* sequencing to evaluate GM samples.

Our analysis comprised 1026 subjects (445 HCs and 581 AD spectrum patients), with a majority of female subjects (59.9%) and a mean age > 65 years. Most studies adjusted for important confounders, including age, sex, education, and body mass index (BMI). The most frequently utilized estimator for alpha diversity was the Shannon index, followed by the Simpson index and Chao1 index, although three studies did not provide information on which index was employed. 12 studies reported beta diversity indices mainly include the Bray-Curtis, weighted and unweighted UniFrac. GM alterations primarily focused on dominant phyla and also different specific bacterial genera or subspecies, such as *A. muciniphila*, *Faecalibacterium*, *Bifidobacterium*, *Bacteroides*, and *Alistipes* (Table 1).

**TABLE 1:** Basic characteristics of the included studies.

References; region	Study design	Adjusted variables	Comparison	AD spectrum group/Control group			Alpha diversity	Beta diversity	Differences of gut microbiota
				Number (n)	Age (years)	Females (%)			
Khedr et al., 2022; Egypt	Case-control	Age, sex, education, smoking, underlying diseases (hypertension, DM, CHD, dyslipidemia)	AD vs. HCs	25/25	68.9 ± 7.5/66.7 ± 8.8	56.0/56.0	NA	NA	①⑩⑪⑫⑮
Sheng et al., 2021; China	Case-control	Sex, education, BMI, presence of APOEε4, emotional state, underlying diseases (hypertension, DM, CHD, dyslipidemia)	SCD vs. HCs	53/38	66.6 ± 6.3/66.7 ± 5.1	81.1/60.5	Chao1, Simpson index, Shannon index	Bray-Curtis, weighted and unweighted UniFrac	④
			CI (MCI and AD) vs. HCs	14/38	73.2 ± 7.8/66.7 ± 5.1	71.4/60.5			④⑨
Liu et al., 2019; China	Case-control	Age, sex, BMI, underlying diseases (hypertension, DM), laboratory tests (hemoglobin, folate, vitamin B12, TT4, TT3)	AD vs. HCs	33/32	74.8 ± 11.3/76.8 ± 9.3	42.4/50.0	ACE, Chao 1, Shannon index, Simpson index	Bray-Curtis, weighted and unweighted UniFrac	②⑭
Zhou et al., 2021; China	Case-control	Age, sex, education, underlying diseases (hypertension, DM, CHD), BMI	AD vs. HCs	60/32	72.8 ± 7.2/71.1 ± 5.9	60.0/56.3	ACE,Chao 1, Shannon index, Simpson index, observed species	Weighted and unweighted UniFrac	⑦⑪⑭
Guo et al., 2021; China	Case-control	Age, education, underlying diseases (hypertension, DM)	MCI vs. HCs	20/18	64.5 ± 4.5/64.2 ± 4.7	80.0/77.8	Shannon index, Evenness, Faith PD	Bray-Curtis, weighted UniFrac	⑤
			AD vs. HCs	18/18	63.5 ± 4.7/64.2 ± 4.7	88.9/77.8			⑤⑩⑫
Liu et al., 2021; China	Case-control	Age, sex, education, BMI, smoking and drinking history, underlying diseases (hypertension, DM), laboratory tests (hemoglobin, folate, vitamin B12, TT4, TT3)	MCI vs. HCs	20/22	68.8 ± 11.2/72.7 ± 8.1	40.0/59.1	ACE, Chao 1, Shannon index, Simpson index	NA	②⑫⑭
Zhuang et al., 2018; China	Case-control	Age, sex, education, underlying diseases (hypertension, DM, CHD, dyslipidemia)	AD vs. HCs	43/43	70.1 ± 8.7/69.7 ± 9.2	46.5/46.5	NA	Weighted UniFrac	⑧⑫
Li et al., 2019; China	Case-control	Age, sex, education, BMI, underlying disease (DM, constipation)	MCI vs. HCs	30/30	65.4 ± 7.6/63.9 ± 5.1	60.0/56.7	Chao 1, Shannon index, observed species	Weighted and unweighted UniFrac	①⑤⑦⑩⑪⑫⑬⑭
			AD vs. HCs	30/30	66.3 ± 5.1/63.9 ± 5.1	50.0/56.7			①⑤⑦⑩⑪⑫⑬⑭
Ling et al., 2020; China	Case-control	Age, sex, BMI, smoking and drinking history, underlying diseases (hypertension, DM, CHD, hyperlipidemia, diarrhea, constipation)	AD vs. HCs	100/71	74.1 ± 9.2/73.1 ± 7.8	57.0/50.7	ACE, Chao 1, observed OTUs, Shannon index, Simpson index	Bray-Curtis, weighted and unweighted UniFrac, Jaccard	①④⑥⑨⑪
Haran et al., 2019; USA	Longitudinal	Age, sex, underlying disease (DM, CKD, cancer), medication history (PPI, statins, antipsychotics, complex drugs), clinical scores (malnutrition, frailty)	AD vs. HCs	21/51	84.7 ± 8.1/83 ± 10.2	83.3/84.3	NA	Jaccard	⑧⑫⑬
Nagpal et al., 2019; USA	RCT	Age, sex, history of alcoholism and head trauma, underlying diseases (COPD, kidney or liver disease, heart disease and mental illness), medication history (statins, hypoglycemic agents)	MCI vs. HCs	11/6	64.3 ± 7.7/65.2 ± 3.7	72.7/66.7	Chao1, Shannon index, observed OTUs, Faith PD	Weighted UniFrac	③⑥
Zhang et al., 2021; China	Longitudinal	age, sex, BMI, smoking and drinking history, exercise	MCI vs. HCs	75/52	62.0 ± 4.1/62.5 ± 4.0	52.0/53.8	Shannon index, Simpson index	Weighted UniFrac	②④⑬
Vogt et al., 2017; USA	Case-control	Age, sex, BMI, race, and diabetes	AD vs. HCs	25/25	71.3 ± 7.3/69.3 ± 7.5	68.0/72.0	Shannon Index, Faith PD	Bray-Curtis, weighted and unweighted UniFrac	③⑥⑪⑫⑬⑭⑮

AD, Alzheimer’s disease; MCI, mild cognitive impairment; HCs, healthy controls; BMI, body mass index; DM, diabetes mellitus; CHD coronary heart disease; COPD chronic obstructive pulmonary disease; CKD chronic kidney disease; TT4, total thyroxine; TT3, total triiodothyronine; PPI proton pump inhibitors; Faith PD, Faith phylogenetic distance; OTUs, operational taxonomic units; NA: not available. ① *A. muciniphila*; ② *Ruminococcus*; ③ *Phascolarctobacterium*; ④ *Faecalibacterium*; ⑤ *Lachnospira*; ⑥ *Dialister*; ⑦ *Lactobacillus*; ⑧ *Lachnoclostridium*; ⑨ *Roseburia*; ⑩ *Prevotella*; ⑪ *Bifidobacterium*; ⑫ *Bacteroides*; ⑬ *Alistipes*; ⑭ *Blautia;* ⑮ *Clostridium*.

### Microbiota diversity in patients with AD spectrum

The richness, evenness, and alpha diversity can all be used to express the diversity of species. Of the 13 included studies, ten studies investigated the bacterial diversity in AD spectrum patients versus HCs, although different indexes were utilized. At the individual study level, results on the difference in alpha diversity between AD spectrum patients and HCs were discrepant: the Shannon index was observed to be significantly lower in patients with AD spectrum compared to HCs in five studies (Vogt et al., 2017; Liu et al., 2019; Ling et al., 2020; Sheng et al., 2021; Zhang et al., 2021), and showed not significant difference in the remaining five studies (Zhuang et al., 2018; Li et al., 2019; Nagpal et al., 2019; Guo et al., 2021; Zhou et al., 2021). Sheng et al. (2021) found on statistically significant reduction in alpha diversity indices for SCD individuals compared to HCs and significantly decreased alpha diversity among CI individuals. Li et al. (2019) found significantly lower diversity (Shannon index) among AD group compared to those of the controls only in blood microbiota samples, but no significant differences among stool microbiota samples. The Simpson index, reported in six studies, was found to be significantly lower in AD spectrum patients compared HCs in four studies (Liu et al., 2019; Ling et al., 2020; Sheng et al., 2021; Zhang et al., 2021) and to be non-significantly different in two studies (Liu et al., 2021; Zhou et al., 2021). Another seven studies used the Chao1 between samples to assess richness and it was found to be significantly lower in AD spectrum patients than HCs in three studies (Liu et al., 2019; Ling et al., 2020; Sheng et al., 2021) and non-significant in four studies (Li et al., 2019; Nagpal et al., 2019; Liu et al., 2021; Zhou et al., 2021).

Beta diversity represents the dissimilarity between the two gut communities. A total of 11 studies (Vogt et al., 2017; Zhuang et al., 2018; Haran et al., 2019; Li et al., 2019; Liu et al., 2019; Nagpal et al., 2019; Ling et al., 2020; Guo et al., 2021; Sheng et al., 2021; Zhang et al., 2021; Zhou et al., 2021) in this review have analyzed the beta diversity of GM, and nine of them consistently identified that the GM of AD spectrum patients showed a remarkably distinct clustering pattern compared with that of HCs (Vogt et al., 2017; Zhuang et al., 2018; Haran et al., 2019; Li et al., 2019; Liu et al., 2019; Ling et al., 2020; Guo et al., 2021; Zhang et al., 2021; Zhou et al., 2021).

### The relative abundance of bacterial genera at different phyla levels

**FIGURE 2:**
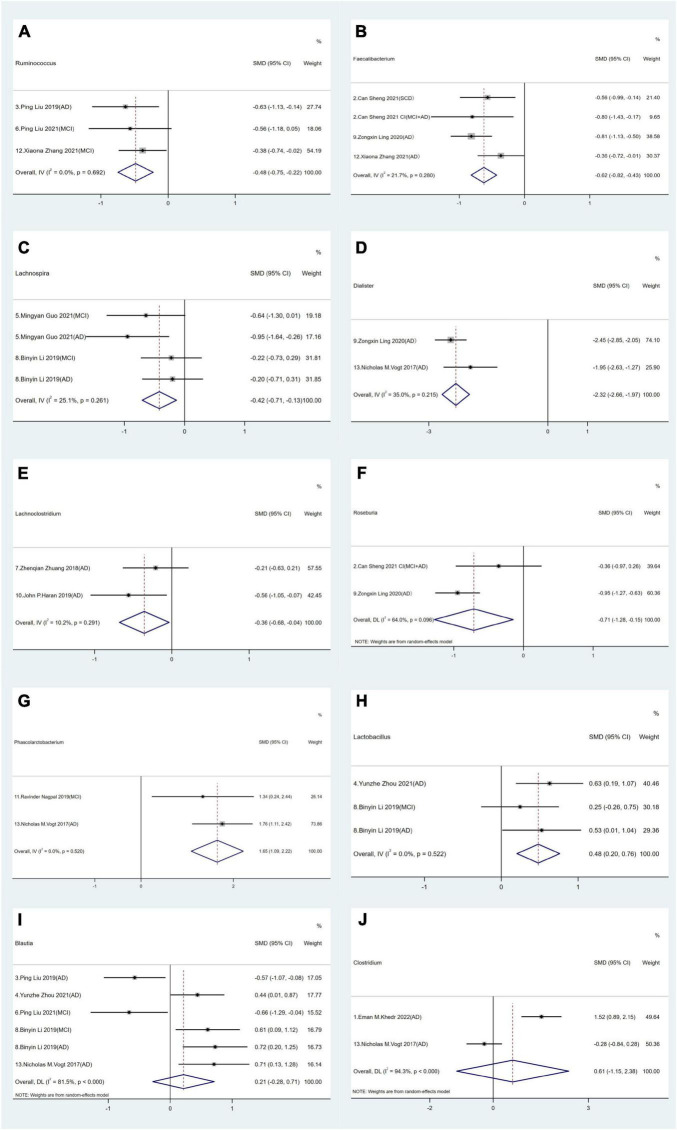
Forest plots of alterations of gut microbiota in the *Firmicutes* phylum compare AD spectrum patients to healthy controls (HCs): **(A)** *Ruminococcus*, **(B)** *Faecalibacterium*, **(C)** *Lachnospira*, **(D)** *Dialister*, **(E)** *Lachnoclostridium*, **(F)** *Roseburia*, **(G)** *Phascolarctobacterium*, **(H)** *Lactobacillus*, **(I)** *Blautia*, **(J)** *Clostridium*.

In terms of relative abundance, *Firmicutes* and *Bacteroidetes* represented the dominant phyla, accounting for 90% of all the GM. *Firmicutes* consists of > 200 different genera comprising Gram-positive aerobic and anaerobic bacteria (Rinninella et al., 2019). Among selected studies, statistical analyses were conducted on 10 distinct genera in *Firmicutes*. We observed that *Ruminococcus*, *Phascolarctobacterium*, *Faecalibacterium*, *Lachnospira*, *Dialister*, *Lactobacillus*, *Lachnoclostridium* and *Roseburia* exhibited distinct intestinal bacterial profiles between AD spectrum patients and HCs. Specifically in AD spectrum patients, a decrease in the relative abundance of *Ruminococcus* (SMD = −0.48, 95% CI: −0.75 to −0.22; *I*^2^_for heterogeneity_ = 0%) (Figure 2A), *Faecalibacterium* (SMD = −0.62, 95% CI: −0.82 to −0.43; *I*^2^_for heterogeneity_ = 21.7%) (Figure 2B), *Lachnospira* (SMD = −0.42, 95% CI: −0.71 to −0.13; *I*^2^_for heterogeneity_ = 25.1%) (Figure 2C), *Dialister* (SMD = −2.32, 95% CI: −2.66 to −1.97; *I*^2^_for heterogeneity_ = 35.0%) (Figure 2D), *Lachnoclostridium* (SMD = −0.36, 95% CI: −0.68 to −0.04; *I*^2^_for heterogeneity_ = 10.2%) (Figure 2E), and *Roseburia* (SMD = −0.71, 95% CI: −1.28 to −0.15; *I*^2^_for heterogeneity_ = 64.0%) (Figure 2F) was observed.

Conversely, an increase in the relative abundance of *Phascolarctobacterium* (SMD = 1.65, 95% CI: 1.09 to 2.22; *I*^2^_for heterogeneity_ = 0%) (Figure 2G) and *Lactobacillus* (SMD = 0.48, 95% CI: 0.20 to 0.76; *I*^2^_for heterogeneity_ = 0%) (Figure 2H) was noted in AD spectrum cases versus HCs. Furthermore, no significant differences in *Blautia* (SMD = −0.21, 95% CI: −0.28 to 0.71; *I*^2^_for heterogeneity_ = 81.5%) (Figure 2I) and *Clostridium* (SMD = 0.61, 95% CI: −1.15 to 2.38; *I*^2^_for heterogeneity_ = 94.3%) were found (Figure 2J).

**FIGURE 3:**
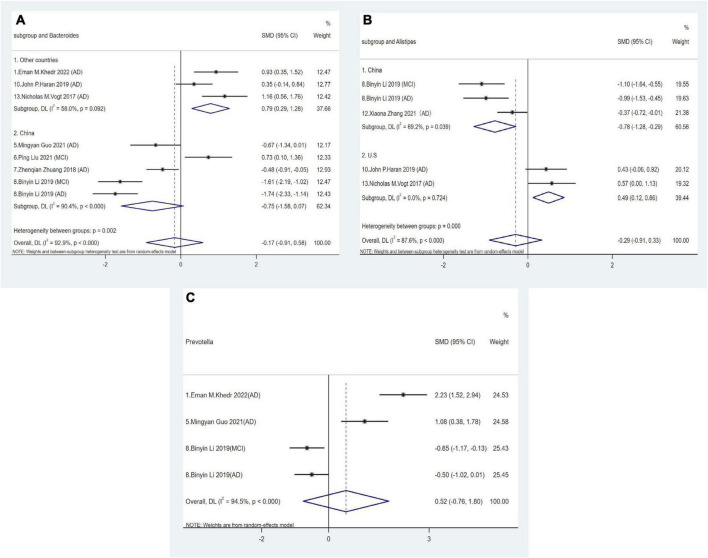
Forest plots of alterations of gut microbiota in the *Bacteroidetes* phylum compare AD spectrum patients to HCs: **(A)** *Bacteroides*, **(B)** *Alistipes*, **(C)** *Prevotella*.

The relative abundance of three genera in the *Bacteroidetes* phylum was assessed in eight studies. The pooled effect size for *Bacteroides* did not exhibit statistical significance when comparing AD spectrum patients and HCs (SMD = −0.17, 95% CI: −0.91 to 0.58; *I*^2^_for heterogeneity_ = 92.9%). However, a subgroup meta-analysis based on geographical location revealed an increased abundance in American and Egyptian cohorts with AD spectrum (SMD = 0.79, 95% CI: 0.29 to 1.28; *I*^2^_for heterogeneity_ = 58%), while no similar trends were observed in Chinese cohorts (SMD = −0.75, 95% CI: −1.58 to 0.07; *I*^2^_for heterogeneity_ = 90.4%) (Figure 3A). Notably, we observed an increased abundance of *Alistipes* across US AD cohorts (SMD = 0.49, 95% CI: 0.12 to 0.86; *I*^2^_for heterogeneity_ = 0%), but a diminished presence in Chinese cohorts (SMD = −0.78, 95% CI: −1.28 to −0.29; *I*^2^_for heterogeneity_ = 69.2%) (Figure 3B). Four cohorts from three studies showed no significant differences in *Prevotella* abundance between AD spectrum patients and HCs (SMD = 0.52, 95% CI: −0.76 to 1.8; *I*^2^_for heterogeneity_ = 94.5%) (Figure 3C).

**FIGURE 4:**
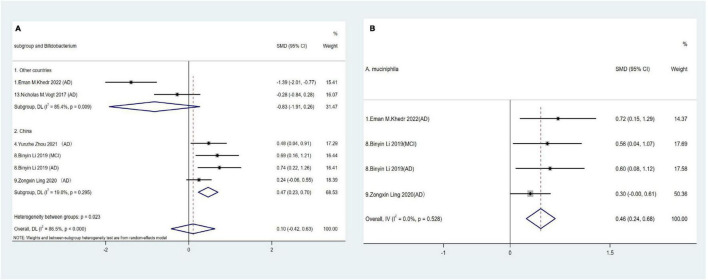
**(A)** Forest plots of alterations of *Bifidobacterium* in the *Actinobacteria* phylum compare AD spectrum patients to HCs. **(B)** Forest plots of alterations of *A. muciniphila* in the *Verrucobacterium* phylum compare AD spectrum patients to HCs.

The *Actinobacteria* phylum is relatively less abundant in elderly individuals and is predominantly represented by the *Bifidobacterium* genus (Vaiserman et al., 2017). However, the pooled effect size for *Bifidobacterium* showed no statistical significance in comparisons between AD spectrum patients and HCs (SMD = 0.1, 95% CI: −042 to 0.63; *I*^2^_for heterogeneity_ = 86.5%). Nevertheless, after adjusting for country, Chinese cohorts revealed a positive correlation between AD and relative *Bifidobacterium* abundance at the genus level (SMD = 0.47, 95% CI: 0.23 to 0.70; *I*^2^_for heterogeneity_ = 19%) (Figure 4A).

*A. muciniphila* is a strictly anaerobic Gram-negative bacterium and the sole representative of the *Verrucobacterium* phylum in human fecal samples (Cani et al., 2022). Three studies across four cohorts showed consistently significant increases in relative *A. muciniphila* abundance in AD spectrum patients (SMD = 0.46, 95% CI: 0.24 to 0.68; *I*^2^_for heterogeneity_ = 0%) (Figure 4B).

### Risk of bias assessments

The critical appraisal of selected studies revealed that for the majority of areas, rigorous methodologies were used. However, some case-controlled studies lacked clear control group selection and provided insufficient detail regarding non-response rates or loss to follow-up (Supplementary Table 1).

**FIGURE 5:**
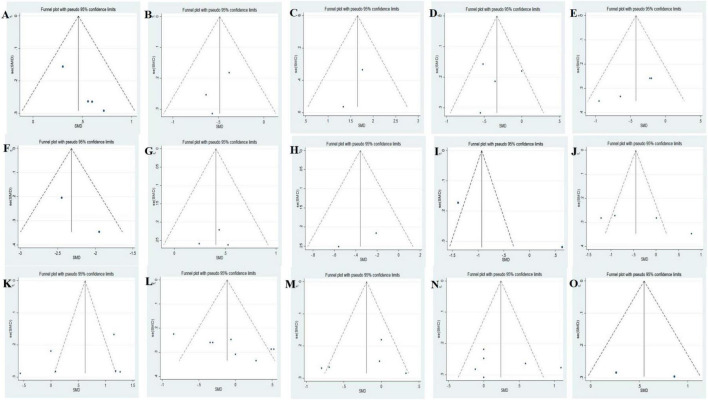
Funnel plots of included studies analyzing alterations of gut microbiota in AD vs. HCs: **(A)** *A. muciniphila*, **(B)** *Ruminococcus*, **(C)** *Phascolarctobacterium*, **(D)** *Faecalibacterium*, **(E)** *Lachnospira*, **(F)** *Dialister*, **(G)** *Lactobacillus*, **(H)** *Lachnoclostridium*, **(I)** *Roseburia*, **(J)** *Prevotella*, **(K)** *Bifidobacterium*, **(L)** *Bacteroides*, **(M)** *Alistipes*, **(N)** *Blautia*, **(O)** *Clostridium*.

Funnel plots were used to evaluate publication bias across indicators. No significant asymmetry was observed in funnel plots, indicating no evidence of publication bias across indicators (Figure 5).

## Discussion

In recent years, both experimental and observational studies have extensively investigated GM alterations in patients with AD, as the identification of such microbial signatures may provide novel insights into AD pathogenesis and potential therapeutic strategies. In this study, we used *16S rRNA* sequencing to explore shifts in GM composition among AD spectrum patients. We observed significant GM composition changes at various taxonomic levels between patients and HCs, and also investigated differential GM abundance in relation to geographical location. Statistically significant lower alpha diversity in AD spectrum patients versus HCs was observed in a substantial proportion of studies, i.e., half for the Shannon index (five out of ten studies), more than half for the Simpson index (four out of six studies), and less than half for Chao1 (three out of seven). Microbial dissimilarities (beta diversity) were observed the gut communities of the two groups.

In the qualitative synthesis, our investigation reveals a statistically significant lower alpha diversity in AD spectrum patients compared with HCs in a substantial proportion of studies, and microbial dissimilarities (beta diversity) between these two groups, were consistently observed across the majority of the studies included, indicating distinct microbial population abundances in the gut of AD spectrum and non-AD spectrum individuals. A previous meta-analysis (Hung et al., 2022) that incorporated eight studies using high-throughput technologies in the 16S rRNA gene showed inconsistent trends in alpha diversity (assessed by the Shannon index and Simpson index) across different stages of AD (MCI and AD), suggesting reduced gut microbiota diversity particularly in the later stages of AD. Another meta-analysis (Jemimah et al., 2023) that re-analyzed he studies included in the Huang Hung et al. (2022) study using QIIME (Quantitative Insights Into Microbial Ecology) software for 16S rRNA gene sequence data yielded results similar to our findings. Among the nine studies included in this analysis, a slight but significant decrease in Shannon diversity was observed, with MCI patients showing increasing trends—although these were non-significant. Moreover, there was stronger consensus in terms of beta diversity, with most studies reporting significant differences among AD, MCI, and cognitively normal cohorts.

In the present systematic review, we found evidence that the composition of GM of AD spectrum persons differs from the non-AD spectrum at the genus level among four phyla. In the quantitative analysis, lower relative proportions of *Ruminococcus*, *Faecalibacterium*, *Lachnospira*, *Dialister*, *Lachnoclostridium*, and *Roseburia* (*Firmicutes* phylum) were found in the AD spectrum group compared to HCs, whereas the genera of *Phascolarctobacterium*, *Lactobacillus* (*Firmicutes* phylum), and *Akkermansia muciniphila* (Verrucomicrobia phylum) were found to be significantly higher in patients. Furthermore, regional variations may have been in play for intestinal microbes such as *Bacteroides*, *Alistipes* (*Bacteroidetes* phylum) and *Bifidobacterium* (*Actinobacteria* phylum).

Mechanistic explanations for changes in GM composition at various taxonomic levels between AD spectrum and non-AD spectrum individuals may be provided by studies investigating the host-gut microbiota relationship through correlating GM composition with circulating metabolites (van der Hee and Wells, 2021). For example, short-chain fatty acids (SCFAs) are generated by gut commensal microbes during indigestible dietary fiber fermentation, including acetate, propionate and butyrate, which possess immunomodulatory potential (Rescigno, 2014). Our study found that almost genera within the phylum *Firmicutes*, such as *Ruminococcus*, *Faecalibacterium*, *Lachnospira*, *Dialister*, *Lachnoclostridium*, and *Roseburia*, are positively correlated with SCFAs (Rescigno, 2014; Sakamoto et al., 2020; Pal et al., 2021; Cai et al., 2022; Ordoñez-Rodriguez et al., 2023), which supports the hypothesis that AD spectrum persons have a distinct microbial profile that is more efficient in fermenting substrates and in producing higher fecal SCFAs concentrations than that of cognitively normal counterparts (Seo and Holtzman, 2024). *Phascolarctobacterium* have been previously reported to be negatively associated with cognitive function indicators, suggesting that mitochondrial damage, calcium homeostasis imbalance, and inflammation may play a role in AD (Ling et al., 2020; Diviccaro et al., 2023; Jemimah et al., 2023). *A. muciniphila* is a mucin-degrading bacterium which uses mucin as its primary energy source; it also exacerbates inflammation by degrading protective mucus layers and exposing immune cells to increased microbial antigen and toxin levels (Jian et al., 2023). In line with our findings, Ling et al. (2020) who used *16S rRNA* sequencing to characterize the GM and observed increased *A. muciniphila* abundance in Chinese- and Kazakh-based AD cohorts. In addition, Li et al. (2019) reported a negative correlation between *A. muciniphila* abundance and Mini-Mental State Examination scores, suggesting potential associations with hippocampal atrophy. Interestingly, contrary to earlier findings, we observed a positive association between *Lactobacillus* species abundance and AD risk in our analyses. Although commonly used as probiotics with beneficial host health effects (Huang et al., 2022), previous studies reported that *Lactobacillus* produced γ-aminobutyric acid (GABA) via glutamate metabolism, which regulated GABA levels in the cerebral cortex and improved cognition, mood, and behavior. In addition, *Lactobacillus* reduced kynurenine concentrations and subsequently enhanced cognitive function in depressed patients (Rudzki et al., 2019). Significantly, a dysregulated kynurenine pathway in the tryptophan metabolic route was proposed as a prominent contributor to AD (Almulla et al., 2022; Liang et al., 2022). However, not all cognitive effects due to *Lactobacillus* are positive. For instance, an increased relative *Lactobacillus* abundance was implicated in obesity and diabetes pathophysiology in both mice and patients (Karlsson et al., 2013; Zeng et al., 2013; Arora et al., 2017). These findings highlight the complexity of GM ecosystem and suggest that a lower relative abundance of the phylum *Firmicutes* and a greater relative abundance of Verrucomicrobia in the AD do not necessarily translate into a common pattern across all genera within these phyla, since several genera from the same phylum may be found in higher or in lower proportions in AD spectrum individuals.

Overall, discrepancies found for regional variations in the abundance of *Bacteroides*, *Alistipes* and *Bifidobacterium* when comparing AD spectrum with HCs may reflect differences in dietary pattern and lifestyle. For example, Dilmore et al. (2023) exploring dietary intervention effects on the microbiome in adults at risk for AD showed that the low-fat American Heart Association Diet increased GABA production in MCI individuals by increasing GABA-producing *Alistipes*. This finding contradicted previous studies reporting dysfunction of GABA signaling aggravating AD pathology and showing lower levels of GABA in postmortem brain tissue from AD patients (Govindpani et al., 2017; Andersen et al., 2022). A previous meta-analysis study has found that an overgrowth of *Bacteroides* in US-based AD cohorts, while this pattern was not observed in Chinese cohorts. The discrepancy might be due to geographical differences in diet, patient populations and in the RNA sequencing area. A large amount of study-specific variation in GM composition and diversity can likely be attributed to interpersonal variability (Ursell et al., 2012). It has been reported that if taxonomic compositions of two individuals’ gut microbiota are quite different, they may still exhibit similar functional activities (Seo and Holtzman, 2024). This current recognition of “functional redundancy” underscores the importance of studying the functional activity and the metabolic potential of GM rather than solely relying on taxonomic composition (Fetzer et al., 2015). Therefore, in order to strengthen the robustness of current evidence, additional individual-level meta-analyses are warranted to elucidate the overall composition, diversity, and stability of the gut microbial network and understand its functional interactions in AD. These analyses should adjust for confounders in a comparable fashion covering critical factors influencing the gut microbiome composition, such as sex, age, diet, physical activity, drug interventions, RNA sequencing area, and standardized microbiome data processing.

Our study strength lies in its comprehensive range of outcomes and rigorous methodology. Furthermore, for better comparability, we only included studies that analyzed the gut microbiome composition by means of high-throughput sequencing techniques. However, several limitations are worth mentioning. First, our study mainly included a cross-sectional design, which only allowed for the identification of associations rather than causal relationships between GM and AD development. Second, despite a thorough and systematic search across databases, selected studies predominantly came from three countries with relatively small sample sizes; thus caution should be exercised when generalizing our findings to other populations. Third, due to limited available study outcomes, we could not analyze dementia subtypes so the impact of GM may vary among different subtypes. Fourth, while we used mature *16S rRNA* sequencing in our study, combining metagenomic sequencing with multiomics approaches could potentially provide more comprehensive and accurate microbial information while minimizing methodological bias. Lastly, although we adjusted for important potential confounders, such as age, sex, BMI, and diabetes, it is important to acknowledge that statistical adjustments may not have completely addressed these issues. Notably, dietary factors were shown to influence GM composition, making it difficult to draw consistent GM profiles in AD patients and even in HCs.

## Conclusion

We systematically evaluated studies investigating altered GM composition using *16S rRNA* sequencing in AD spectrum patients when compared to HCs. In summary, a reduced microbiota diversity and a significantly distinct pattern of clustering with regard to beta diversity were observed in AD spectrum patients when compared with those in HCs. Additionally, consistent with previous findings, patients with AD spectrum exhibited significant intestinal flora changes. Specifically, decreased *Ruminococcus*, *Faecalibacterium*, *Lachnospira*, *Dialister*, *Lachnoclostridium*, and *Roseburia* abundance was recorded in patients while *Phascolarctobacterium*, *Lactobacillus*, and *A. muciniphila* were more enriched when compared to HCs. No significant differences in the relative abundance of other microbial taxa were identified. Furthermore, regional variations may have been in play for the GM, such as *Bacteroides*, *Bifidobacterium*, and *Alistipes*. Currently, debates are ongoing regarding GM pathogenicity and associated metabolites; therefore, well-designed studies using innovative approaches are required to gain a better understanding of the GM in AD development. Such insights may pave the way for novel GM-based strategies to prevent and treat AD.

## Data availability statement

The original contributions presented in this study are included in this article/Supplementary material, further inquiries can be directed to the corresponding authors.

## Author contributions

HL: Data curation, Formal analysis, Investigation, Methodology, Project administration, Software, Visualization, Writing – original draft. XC: Data curation, Software, Visualization, Writing – original draft. YL: Methodology, Software, Supervision, Validation, Visualization, Writing – original draft. FH: Formal analysis, Investigation, Project administration, Visualization, Writing – original draft. AT: Conceptualization, Supervision, Writing – review & editing. RZ: Conceptualization, Project administration, Supervision, Writing – review & editing.

## Footnotes

1
https://doi.org/10.37766/inplasy2024.6.0067
