# Supplementary material for: Gut microbiota changes in patients with Alzheimer’s disease spectrum based on 16S rRNA sequencing: a systematic review and meta-analysis
Source: Front Aging Neurosci. 2024 Aug 8;16:1422350. doi: 10.3389/fnagi.2024.1422350 (PMC11338931; doi:10.3389/fnagi.2024.1422350)
Supplement: Supplementary file 1 [file Data_Sheet_1.PDF]

S1 Table. Quality assessment using Newcastle-Ottawa quality assessment scale for the observational studies included in the meta-analysis.

study	Selection				Comparability	Exposure			
	Adequate definition of cases	Representativ eness of cases	Selection of controls	Definition of controls	Control for important factors*	Ascertainment of exposure	Same methods to ascertain for cases and controls	Non-response rate	Total Score
Khedr 2002	★	★		★	★★	★	★		7
Sheng 2021	★	★		★	★★	★	★		7
Liu 2019	★	★		★	★★	★	★		7
Zhou 2021	★	★		★	★★	★	★		7
Guo 2021	★	★		★	★★	★	★		7
Liu 2021	★	★		★	★★	★	★		7
Zhuan 2018	★	★	★	★	★★	★	★		8
Li 2019	★	★		★	★★	★	★		7
Ling 2020	★	★		★	★★	★	★		7
Haran 2019	★	★	★	★	★★	★	★		8
Zhang 2021	★	★	★	★	★★	★	★		8
Vogt 2017	★	★	★	★	★★	★	★		8

*A maximum of two stars can be allotted in this category, one for Age and Sex, the other for other controlled factors.
